# Bruises of the Lungs

**Published:** 1895-10

**Authors:** John Parmenter

**Affiliations:** Professor of anatomy and adjunct professor of clinical surgery, Medical Department, University of Buffalo


BUFFALO MEDICAL JOURNAL.

VOL. XXXV. OCTOBER, 1895. No. 3.

### Original Communications.

### BRUISES OF THE LUNGS.

By JOHN PARMENTER, M. D.,
Professor of anatomy and adjunct professor of clinical surgery, Medical Department, University of Buffalo.

I HAVE been led to select this subject for the reason that the profession has not as clear an understanding of the mechanism and symptomatology of contusions of the lung as the frequency and importance of the condition demands. Our text-books, too, are quite silent upon the subject. We hear constantly of contusions and lacerations of the brain and abdominal viscera, and have a fairly definite idea of them; but, for some reason or another, this knowledge has not extended to similar conditions affecting the lungs. In what I shall have to say upon this subject, I wish it understood that only such cases are in mind in which the thoracic walls remain intact; that is to say, no wound of the thoracic wall together with fracture or dislocation of the ribs; cases, therefore, in which there is no open communication between the point of impact of the acting force and the lesion in the lung.

One is apt to think lightly of a blow upon the chest which inflicts no injury to the chest wall, but when we remember that cases are recorded in which almost no organ has escaped bruise or laceration, including even the pericardium, heart, aorta and esophagus, we are reminded that much serious injury may follow what seems to be a trivial cause.

Before passing to the clinical side of our subject, let us dwell for a short time upon the mechanism and pathological anatomy of this class of injuries. Why does a blow upon the thorax, the chest-wall remaining intact, cause a bruise or laceration of the lung? The chief factor in the production of this kind of injury is, unquestionably, the elasticity of the thorax wall. Under the pressure of the acting force it is driven against the underlying lung and then rebounds. The amount of elasticity varies with the age, and the like, of the individual and, hence, the degree of force necessary to produce a certain effect also varies. This elasticity alone, however, does not explain why an elastic and mobile organ like the lung is so easily contused. Gosselin, who has added so much to our knowledge of lung injuries, explains the bruises in this way: when a person is about to be hit, by instinctive action he holds his breath, so that, with the glottis closed, the air imprisoned in the lung cannot find exit at the time the blow is received and the lung is to all intents and purposes a solid body, giving resistance to the acting force. This it does not withstand, and ruptures either at the point of impact or at some weak point.

The theory of Peyrot is, to my mind, a more satisfactory and rational one. This author believes that we may regard the lung, throughout its whole external surface, as adherent to the thorax wall by virtue of the pleural space. If a blow infringes upon a point of the thoracic wall, it depresses the same and successively the parietal and the pulmonary layers of the pleura. The remainder of the lung, from its natural adherence to the wall, does not follow the movement. If, now, a part, but little tense naturally, is straightened out under the force, it ruptures. In this way a direct rupture occurs. Indirect ruptures are less easily explained. Large contusions cause more or less pronounced changes in form, so that parts of the lung normally adherent to the thoracic wall tend to become separated from each other. In this way traction is made upon the pulmonary tissue, which results in a tear, the seat of which may be quite distant from the point upon the thoracic wall at which the force acted.

It is not unlikely that when the lung is filled with air it is more easily ruptured, as Gosselin maintains, than when empty; but, in addition, it must be remembered that during effort the lung is also fuller of blood and the delicate membranes in a condition of tension, which makes them more easily ruptured. Other predisposing causes of rupture are tubercular infiltration, tubercular cavities, emphysema, and pulmonary congestion such as occurs in cardiac disease, chronic alcoholism, and the like.

One other fact in the mechanism of these injuries must not be overlooked, and that is, that bruises or lacerations are occasioned in the same way, whether the ribs or costal cartilages are broken or not, the actual tearing of lung substance by the end of a broken rib excepted. Normally the two layers of the pleura lie in intimate contact with each other. This contact is maintained throughout all phases of the respiratory movements in the fullest inspiration or deepest expiration. When a collection of fluid (blood, serum or pus,) occurs between these pleural layers, a space is formed at the expense of the lung, which now loses its power of expansion in proportion to the size of the space or its contents, which is the same thing. This may go on until the whole pleural cavity is filled with fluid, when naturally the lung has become so compressed that it receives no more air and respiration on the affected side is inhibited. The above mentioned space may be occupied by still another foreign substance, and that is air. In cases like those under consideration, where no external wound leads to the injured lung, air can enter the pleural space by passing from a bronchiole through a laceration in the lung and pulmonary pleura, which communicates with the pleural cavity, of course. The lung immediately collapses and the air in the lung is forced into the same space. Inspiration simply forces more air into the constantly increasing space, until finally the patient becomes nearly or entirely suffocated from pressure upon the contents of the thorax.

When the two layers of the pleura are adherent, the lung cannot collapse, but allows the inspired air to pass through it into the tissues just outside of it, when we have the condition known as emphysema. Of this symptom more will be said later on.

Jobert makes three degrees of bruises:

*First degree.*—The lung shows small hemorrhagic punctata, resulting from rupture of the capillaries, but the pulmonary tissue itself is not torn.

*Second degree.*—Beneath the healthy pleura the lung tissue is found to have suffered small ruptures extending to the alveoli and bronchioles, including the vessels about them. From the rupture of the blood-vessels there results small depots of blood scattered here and there.

*Third degree.*—This includes extensive tears of the lung, bronchi and larger vessels. Bruises of the first and second degrees are usually recovered from. When death does follow, it is due to inflammatory troubles consequent upon the original injury.

In injuries of the third degree, patients often die very rapidly. The bruises and tears may be of any degree, from slight to those involving the whole lung. There may be only one or many. They may occur in any portion of the lung.

Certain pathological conditions follow bruises of the lung, which need especial emphasis, as they have an important bearing upon the symptomatology and treatment of the same. These are:

*Hemothorax.*—This occurs frequently. The blood, as before said, is poured out into the pleural space, and its presence there means rupture of the pleura and lung substance and the tearing through of one or more pulmonary vessels.

*Pneumothorax.*—Pneumothorax is caused in precisely the same way as hemothorax, except that the bronchioles instead of the vessels are the seat of rupture, and air instead of blood is forced into the pleural cavity. As a matter of fact, the two conditions, hemo- and pneumothorax commonly coëxist.

*Emphysema.*—This results from the combination of a pneumothorax with the wound of the inner wall of the thorax, through which the air in the pleural cavity presses into the tissue underneath the skin covering the chest. It is especially apt to occur in restless patients, where, in holding the breath in anticipating pain, the intrapleural pressure is increased and the contained air driven into the subcutaneous tissues. This continues until the extra- and intrapleural pressure are the same. The emphysema may extend over almost the entire body and cause very remarkable effects in the personal appearance of the patient.

The emphysema may finally extend to the mediastinum and under the pleura of the sound side, when fatal dyspnea may, and often does, ensue. Emphysema may appear immediately after the injury has been received. Under these circumstances it is apt to be speedily fatal. It is due to injury to the root of the lung, that is to say, to rupture of a bronchus, whereby air is immediately forced into the mediastinal space, and thence upward into the neck and face, and exercises deadly pressure from the very beginning.

*Symptoms.*—These, naturally, vary with the degree of the injury present. In the milder forms of injury, the symptoms are often few and but little pronounced, being limited to a slight dyspnea, increased respiratory frequency, a little pain and somewhat bloody expectoration. Even one or more of these symptoms may be absent, and the diagnosis only be made certain by the appearance of complications which may arise some time after the receipt of the injury.

But let us suppose a graver case. Here we shall find our patient in greater or less shock or even collapse, face pale, pulse small and compressible, respiration short and labored, with marked dyspnea. Profuse hemoptysis is present. Physical examination of the lung reveals sonorous râles, with absence of vesicular murmurs, but with amphoric breathing and occasional metallic tinkling. If to these symptoms we add the enlargement of the injured side, we have a combination of symptoms and signs which indicate the existence of a pneumothorax. This condition may be absent, however, in severe cases where the rupture occurs in and is confined to the central parts of the lungs, under which circumstance the physical signs and symptoms will be those of a cavity.

Emphysema, particularly at the base of the neck, soon appears for reasons already given, and finally in some cases a more or less pronounced hemothorax can be demonstrated. A sign upon which considerable stress has been laid by some authors (notably Morel-Lavallee and P. Reynier,) is that called the *bruit de roue hydraulique* or *de roue de moulin*. It consists of a series of splashing sounds heard in the precardial region and synchronous with the heart-sounds. They resemble those due to the shaking together of air and water and may be so pronounced as to be heard at some distance from the patient. It is apt to disappear when the patient assumes the sitting posture. It is due to the agitation of a collection of air and fluid by the movements of the heart. Such conditions are often fulfilled in hemo-pneumothorax. It is not necessarily a grave symptom and disappears after a few days, as the air becomes absorbed.

COMPLICATIONS.

*Bronchitis or Broncho-pneumonia.*—This complication appears early; that is, in the first week. It may be very severe and continue for many days—twenty or more.

*Traumatic Pneumonia.*—This is supposedly induced by an infection which the lung incurs by the bruise. The agent is the pneumococcus of Friedländer. It appears usually between the third and fourth days. It has not the violent onset of a typical frank pneumonia, but begins without any initial chill, in a blind and insidious way. The temperature rarely runs high, seldom exceeding 40 degrees C. It may be benign in character and terminate in from eight to ten days; nevertheless it is often fatal.

*Gangrene.*—This occurs from an intense inflammation in the contused area. It may be rapid, but is usually quite slow in its course. Cases have been reported where portions of the lung tissue have been expectorated in a way suggestive of the falling off of a dry scab. Now and then the gangrene becomes general and involves the entire lung.

*Pleurisy.*—Pleurisy is the most common complication of a bruise of the lung. It may assume various forms and degrees. It may be mild with small effusion, running an ordinary course with ultimate absorption of the fluid, or it may be severe, with an effusion so great as to require tapping. The effusion may become purulent, making a veritable empyema. Putrid pleurisy occurs in certain cases where hemo-pneumothorax is present and, of course, constitutes a grave complication.

*Prognosis.*—The termination of lung contusions is, naturally, dependent upon the degree of injury and the complications which follow. In general, we may say that recovery follows in almost all cases, even when hemo- and pneumothorax are present, provided the lesions remain aseptic, and they usually do. The course of the convalescence is simple and short, with but little connective tissue formation, and this limited to the immediate region of the injury. Infection through the trachea and bronchi occurs only seldom ; but, when it does take place, a purulent pleurisy results, usually severe in character and often fatal.

Given a patient who has escaped death from rupture of some large vessel or some of the important thoracic viscera, our prognosis will depend upon the nature and severity of the inflammatory complications, as above enumerated. These are usually due to infection, and what is vitally important, from the prognostic point of view, for us to remember is that these complications, in their gravest forms, may follow an injury in which the evidences of bruise or laceration of the lung, at the time of accident, were so slight as to render the diagnosis difficult and uncertain.

Furthermore, the existence of certain conditions, such as diabetes, alcoholism, hepatic and renal disease, must be taken into account, inasmuch as they predispose to pulmonary suppuration.

Do bruises of the lung favor the development of pulmonary phthisis ? Mendelsshon has published seven cases in which he believes consumption to have been given an impetus in this way. There seems no reason why, in subjects predisposed to tuberculosis, a bruise or laceration may not become the starting point of tubercular infection, just as it certainly does in other portions of the body.

This is an interesting and important question which only the future will determine accurately for us; it, however, needs the careful consideration of the general practitioner and the recording of accurate cases calculated to throw light upon the subject.

### TREATMENT.

When the injury is of the graver form, shock, pain, dyspnea and hemorrhage will be the more urgent symptoms that demand relief. For shock, judicious stimulation with hypodermatic injection of brandy and ether, warmth to the body, and the like, will give the best results.

Pain and dyspnea are best mitigated by morphia subcutaneously, in appropriate doses. It often acts in a magical way. Pain is relieved and almost immediately the breathing becomes quiet and regular and the tension in the thorax cavity speedily lessened. Cold in some form or another applied to the chest wall aids in lessening the pain, controlling the hemorrhage and retarding or preventing further pneumothorax and emphysema.

From the very beginning, in even the mild cases, the patient should remain in bed and not be allowed to speak except in monosyllables, and sparingly in these. By such treatment the complications will be lessened, if not prevented, for, as we have already seen, the mildest cases are often succeeded by grave sequelæ. It is not unlikely that negligence of the simple law of rest may frequently be the cause of the serious sequences so often observed.

*Hemorrhage.*—When it continues and threatens the life of the patient, of course it demands immediate attention. In addition to other measures already recommended, anto-transfusion may be employed with advantage. If bandages are not immediately at hand, as they usually are not, the patient's legs and arms should be elevated until bandages can be applied. The question has been many times raised, whether a more radical treatment for hemorrhage should be instituted, inasmuch as in severe bleeding conservative measures oftentimes seem of little or no value. In other words, are we justified in opening the thorax and searching directly for the bleeding point or points? When we reflect that to locate the source of hemorrhage and to control it when found (if the injured vessel be a large branch of the pulmonary artery) belongs to the class of impossibilities almost, it will, I am sure, be a long time before surgeons will recommend and practise such radical treatment. If, however, the hemorrhage assumes such proportions as to cause threatening symptoms from pressure, namely, dyspnea and cardiac embarrassment, it is imperative to relieve the condition. This can be safely done by withdrawing, through a medium-sized trocar, as much blood as is necessary to relieve pressure. It is needless to say that this procedure must be conducted under the strictest antiseptic precautions. It often happens that the blood becomes clotted and will not pass through the canula, when we may incise the thorax wall (just as in the operation for empyema) and turn out the necessary amount of blood. The wound should then be thoroughly approximated and an antiseptic dressing applied. Unfortunately this treatment does not always succeed, for upon withdrawal of the blood the pressure upon the ruptured vessel is relieved and the hemorrhage begins anew. The procedure is, therefore, one to be employed only when other treatment has failed and the life of the patient is in imminent jeopardy.

It not unfrequently happens that severe pressure symptoms are caused by air (pneumothorax). When such is the case, paracentesis affords a safe and effectual method of treatment. This may be repeated as often as the pressure symptoms recur, as will be the case until the wound in the lung and pulmonary pleura becomes closed. The danger in severe cases of pneumothorax of pneumonia setting in as a result of the pressure has led some surgeons to practise incision and drainage of the pleural cavity. This, when done antiseptically, is not at all dangerous. It wards off the pneumonia and hastens the closure of the wound between the pleural space and the ruptured bronchiole.

Emphysema sometimes assumes proportions that make it demand surgical intervention. Here the best thing to be done is to make long and deep incisions in different parts of the swollen area, and by judicious pressure of the fingers from the circumference of the area toward the incision opening, to press out the air contained in the tissues.

The treatment of empyema, when it occurs, and of the various other complications already mentioned, will naturally be the same as when they are due to the more common causes. The limits of this paper preclude any further remarks concerning them, nor is it necessary to do so.

519 FRANKLIN STREET.

